# Novel Phospholipid-Protein Conjugates Allow Improved Detection of Antibodies in Patients with Autoimmune Diseases

**DOI:** 10.1371/journal.pone.0156125

**Published:** 2016-06-03

**Authors:** Simone V. Samuelsen, Arindam Maity, Mads Nybo, Claudia Macaubas, Lars Lønstrup, Imelda M. Balboni, Elizabeth D. Mellins, Kira Astakhova

**Affiliations:** 1 Department of Physics, Chemistry and Pharmacy, University of Southern Denmark, Odense, Denmark; 2 Department of Clinical Biochemistry and Pharmacology, Odense University Hospital, Odense, Denmark; 3 Department of Pediatrics, Program in Immunology, Stanford University, Stanford, California, United States of America; 4 Department of Business and Economics, University of Southern Denmark, Odense, Denmark; Renal Division, Peking University First Hospital, CHINA

## Abstract

Reliable measurement of clinically relevant autoimmune antibodies toward phospholipid-protein conjugates is highly desirable in research and clinical assays. To date, the development in this field has been limited to the use of natural heterogeneous antigens. However, this approach does not take structural features of biologically active antigens into account and leads to low reliability and poor scientific test value. Here we describe novel phospholipid-protein conjugates for specific detection of human autoimmune antibodies. Our synthetic approach includes mild oxidation of synthetic phospholipid cardiolipin, and as the last step, coupling of the product with azide-containing linker and copper-catalyzed click chemistry with β2-glycoprotein I and prothrombin. To prove utility of the product antigens, we used enzyme-linked immunosorbent assay and three cohorts of samples obtained from patients in Denmark (n = 34) and the USA (n = 27 and n = 14). Afterwards we analyzed correlation of the obtained autoantibody titers with clinical parameters for each patient. Our results prove that using novel antigens clinically relevant autoantibodies can be detected with high repeatability, sensitivity and specificity. Unlike previously used antigens the obtained autoantibody titers strongly correlate with high disease activity and in particular, with arthritis, renal involvement, anti-Smith antibodies and high lymphocyte count. Importantly, chemical composition of antigens has a strong influence on the correlation of detected autoantibodies with disease activity and manifestations. This confirms the crucial importance of antigens’ composition on research and diagnostic assays, and opens up exciting perspectives for synthetic antigens in future studies of autoimmunity.

## Introduction

Autoimmune diseases are very diverse and abundant (>80), and they are in general characterized by production of antibodies against one’s own biomolecules and tissues [1]. The causes of autoimmune disease are not fully understood [1]. However, several conditions are characterized early on by production of autoimmune antibodies (autoantibodies) against cellular phospholipids and phospholipid-protein conjugates [2]. At present, only the first type of autoantibodies are investigated, but the latter could also become a valuable biomarker in diagnosis and monitoring of autoimmune diseases, including antiphospholipid antibody syndrome (APS) and systemic lupus erythematosus (SLE). Multiple reports confirm high clinical relevance of autoantibodies towards phospholipid-protein complexes but not towards phospholipids alone [3]. This is due to the fact that autoantibodies do not recognize a phospholipid itself but its complex with numerous plasma proteins [3]. Examples include autoantibodies produced against non-covalent complexes of the phospholipids cardiolipin and phosphoethanolamine with β2-glycoprotein I (β2GPI) and prothrombin. These autoantibodies are hallmarks of APS and are also observed in patients with SLE and autoimmune neurological diseases [3,4]. If not early diagnosed and treated, these conditions can cause serious health complications and even death [5].

The incidence of SLE in the general US population is approximately one in 2000, with a nine-to-one female gender prevalence, occurring mostly in non-Caucasian subjects [6]. However, there are still no definite diagnostic criteria, monitoring approaches or treatments. In particular, SLE patients are very diverse with respect to disease manifestations and clinical parameters, which have to be taken into account [7]. Therefore, reliable autoantibody tests with synthetic antigens could become an important component of diagnostics, research and point-of-care monitoring of autoimmune diseases.

**Fig 1:**
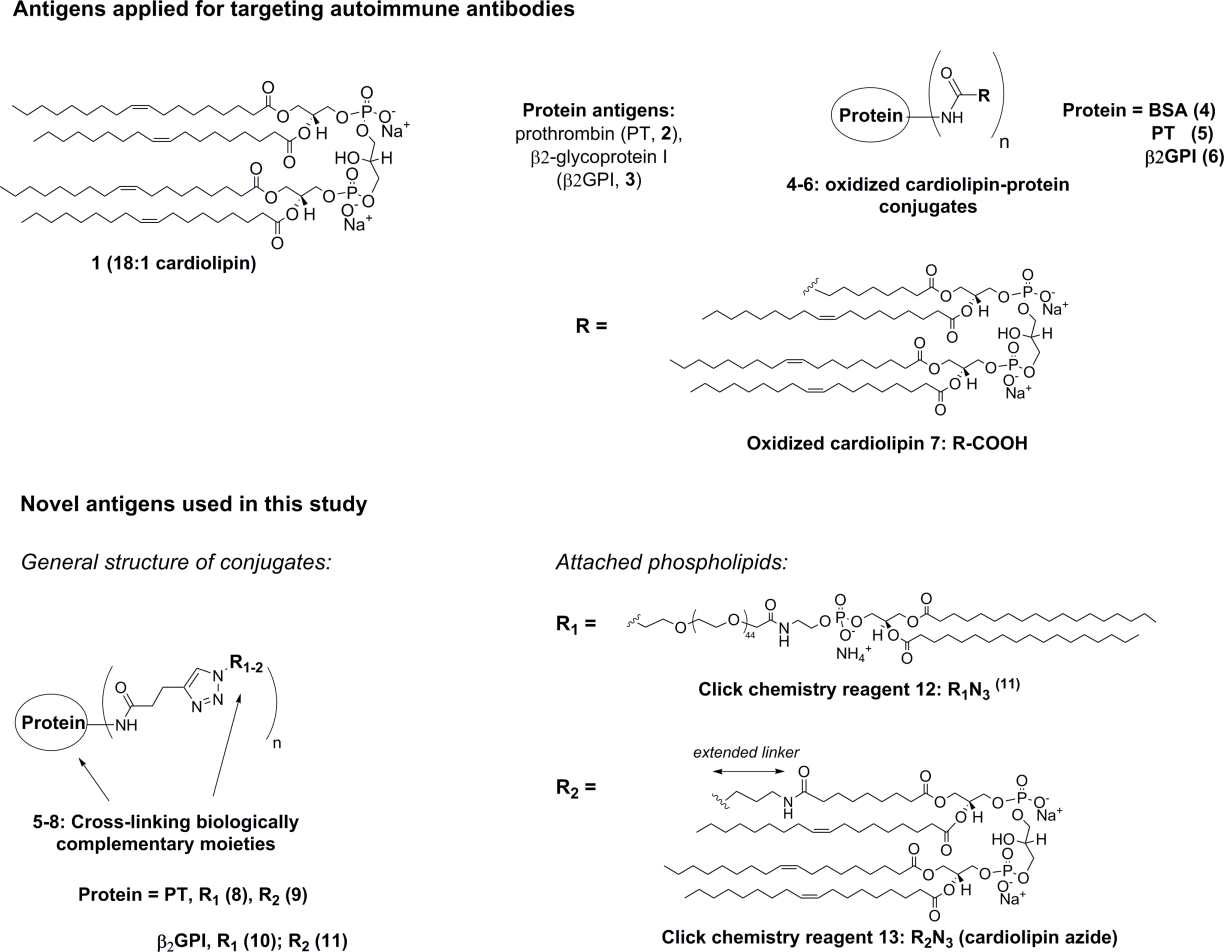
Chemical structures of previously reported antigens and new molecules. Previously, cardiolipin was subjected to harsh oxidation prior to coupling to protein cargos via amide bond (compounds **4**–**6**). Herein, linker between cardiolipin and proteins has been increased, and biologically complementary prothrombin and β2GPI were attached to azide **13** under mild conditions.

Multiple reports confirm biological activity of phospholipid-protein complexes and their role in autoimmunity. However, most diagnostic assays have applied lipids and corresponding proteins separately (Fig 1) [8], because synthesis of conjugates with well-defined structure and purity was previously unachievable. Moreover, phospholipid−autoantibody binding is highly sensitive to the assay conditions including temperature of incubation and exposure to light [8–10]. These factors likely contribute to low reproducibility in assays and to weak correlation of the results with clinical manifestations of the disease. Applying lipids and proteins as separate antigens is due to the previously unachievable synthesis of synthetic conjugates with well-defined structure and purity. Indeed, lipidation of proteins is a challenging chemical process, which potentially can result in low yields and insufficient purity of products [9]. Recent progress in bioconjugation, including the development of copper-catalyzed azide-alkyne cycloaddition, or CuAAC click chemistry, opens up an exciting opportunity to create such reagents using synthetic biomolecules [11]. Advantages of the synthetic antigens compared to natural analogues are high chemical and biological stability, purity and well defined chemical structure of the bioconjugation products [11]. Recently, we proved high efficacy of the CuAAC approach for the synthesis of phosphoethanolamine conjugates with prothrombin and β2GPI [9]. However, attempts to attach oxidized cardiolipin **7** to same proteins resulted in low yields and low biologic activity of products (Fig 1, compounds **4–6**). Clearly, the conditions of oxidation for cardiolipin have to be optimized in order to obtain desired antigens in high yields [9].

Cross-reactivity of autoantibodies is another obstacle to their utilization as biomarkers for autoimmune conditions [9]. Low specificity results in low reliability of tests and limits their usefulness in diagnostics and understanding autoimmune diseases [9,12,13]. In particular, cross-reactivity with nucleic acids and other proteins has been reported [9,12]. We hypothesize that this is caused by high heterogeneity of currently applied antigens, absence of complementary protein within the molecular structure and low chemical stability.

In this paper, we introduce novel synthetic antigens, cardiolipin-β2GPI and cardiolipin-prothrombin, and prove their utility in diagnostics of autoantibodies. Novel antigens reported herein are prepared by convenient click chemistry approach in high yields and purity. Tests of specificity against human monoclonal antibodies prove our hypothesis of improved specificity as a result of conjugating biologically complementary molecules in a regioselective fashion. The developed synthetic conditions take into account the following challenges of protein lipidation: 1) sensitivity of lipids to oxidation; 2) high affinity of lipids to plastic; 3) steric hindrance of lipids and proteins which decreases cross-coupling yields. Next, we show the utility of the prepared phospholipid-protein conjugates in enzyme-linked immunosorbent assay (ELISA) using two different cohorts of patient samples and healthy controls. Finally, we investigate correlation of the observed autoantibodies with multiple clinical parameters and verify the causal effect of several disease manifestations on the elevated autoantibody titers.

## Materials and Methods

18:1 cardiolipin **1** (1ʹ,3ʹ-bis[1,2-dioleoyl-*sn*-glycero-3-phospho]-*sn*-glycerol (sodium salt)) and phosphoethanolamine azide **12** (distearoyl-sn-glycero-3-phosphoethanolamine-*N*-[azido(polyethulene glycol)-2000] (ammonium salt)) were purchased and used as received from Avanti Polar Lipids. β2-Glycoprotein I (β2GPI) and prothrombin were obtained from Diarect Antigens and Sigma. Pentynoic sulfotetrafluorophenyl (STP) ester, 3-azidopropan-1-amine, reagents and solvents for click chemistry were provided by Lumiprobe. Other reagents, buffers and solvents were obtained from Sigma Aldrich and used without additional purification. Synthesis of azide **13** and conjugation to proteins is described in S2 Appendix.

All the reactions involving phospholipids and storage of their conjugates with proteins were performed in glass vials and in dark. 96-well Maxisorb NUNC microplates for ELISA assays were purchased from Thermo Fisher Scientific.

Human monoclonal antibodies used as controls for unspecific binding were purchased from NIH AIDS Reagent Programme (a-p24, a-gp41) or provided by Stanford University (B12; produced in Mellins lab) [14,15]. Catalogue numbers: a-p24, 530; a-gp41, 531. Host species for the antibodies were humans. Antigens used to raise and test antibodies: p24, gp41 and gp120. Final antibody dilution was 1:10.000. Each antibody in a dilution 1:10.000 was validated by indirect ELISA assay with corresponding antigen: p24, gp41 and gp120.

Bradford assay was performed following manufacturers protocol using standard reagents (BioRad) [16].

Human samples were obtained from Immunovision, USA (HNP: human normal plasma from a healthy adult patient, HCL: human disease stated plasma from a patient diagnosed with APS), Stanford University Hospital, Stanford, CA, USA (sera, SLE subjects and matched healthy controls), and Odense University Hospital, Odense, Denmark (plasma). Several patients at Stanford University Hospital provided multiple samples in different time points. This resulted in a total number of 40 and 16 samples for SLE and healthy controls, respectively. The samples were characterized by clinical data, common serological biomarkers, treatment intensity and diseases activity stage. Samples were obtained under IRB approved protocols. Written approval by The Danish Data Protection Agency was obtained in November 2015. Committee of Information Safety of the Region Southern Denmark at the Danish Data Protection Agency specifically approved the whole study (permission signed by Pernille Winther Christensen).

Age, gender, race and ethnicity of subjects have an influence on the origins and outcome of autoimmune diseases and therefore were determined after the establishment of diagnosis. This was done following National Institutes of Health (NIH) guidelines and taking into account most relevant grouping to studies of autoimmune diseases [6]. Based on previous literature, groups were formed as follows: male/female; age at diagnosis/age at sample collection; Hispanic and non-Hispanic; White, Black, Asian, and Hawaiian/Pacific Islander.

No additional rules for human categorization were required by funding agency. Confounding variables such as socioeconomic status, nutrition, environmental exposures were not used in this study. Further details on characterization of patient samples are given in S3 Appendix.

ELISA assays, reproducibility analysis and testing the stability of antigens were carried out as described before [9]. Initially, background signal of each sample was tested to be below A_450_ 0.06 using non-coated microplates upon blocking at the same conditions as for the original experiment. To determine equilibrium time for the antigen binding, pre-coated microplates were subjected to incubation with samples in dilution 1:200 over a time course of 40 min– 3 hrs. Secondary incubation was always carried out for 1 h at room temperature using 1:35.000 dilution of corresponding HRP-conjugate. Results of this assay are presented in the S2 Appendix.

Linear range for each antigen was determined by testing series of control dilutions (HNP, HCL in dilutions 1:50 to 1:2000). According to the results dilutions 1:100–1:750 were within the linear range of assay for each antigen (R^2^ > 0.97). For the controls and patient samples, weak positive (+/-) and positive (+) signal were determined as 2- and 3-fold signal, respectively, above the mean value for a healthy control cohort (n = 14) [17].

For the dose response assay, 8 samples (30%, Stanford University Hospital SLE cohort) were randomly selected. Next, for each applied antigen, absorbance (A_450_) values were determined by ELISA in 1:100 serum dilutions and converted to U/mL values equation dividing the obtained absorbance at 450 nm to the total sample protein (in g/mL).

Having done this, dilution of each serum was normalized to fit the following titration (Unit/mL *10^−3^): 0.04, 0.07, 0.11, 1.5, 2.9, 4.5 and 6.7. Finally, ELISA assay was repeated for each antigen using selected sera and these 7 dilutions.

For statistical analysis, the following clinical parameters of each patient were considered: age (at onset and at sample date), gender, ethnicity, race, diagnosis, disease activity (at onset and at sample date), clinical manifestations, patient’s complaints, and applied medications which are known to induce autoantibodies (see S3 Appendix). The latter formed eight groups: corticosteroids, hydroxychloroquine, atorvastin, simvastin, corticosteroids+hydroxychloroquine; hydroxychloroquine+atorvastin, atorvastin+simvastin; corticosteroids+simvastin. Differences were analyzed for statistical significance with multiple regressions (ordinary least squares (OLS) analysis) in Stata [18]. Groups were compared for difference in means of antibody titers using ANOVA. Holm–Bonferroni method was used to counteract the problem of multiple comparisons [18]. A p-value (regular/adjusted) of less than 0.05 was considered significant for each correlation. Statistical power of the corrected test for diverse medication (eight comparisons in total) was estimated to be 77% for a total sample size 74 and anticipated effect size ≥ 0.2 (calculated in Stata) [19].

## Results

### Synthesis of novel antigens

Initially, we selected cardiolipin-β2GPI and cardiolipin-PT as target molecules for this study, owing to their documented biological activity and relevance to SLE and APS (Fig 1) [4,9]. Previously, we and others have reported synthesis of phospholipid-protein conjugates using oxidized cardiolipin RCOOH [9]. Product carboxylic acid was used for coupling with amino groups of proteins yielding antigens **4–6** (S2 Appendix) [9]. However, we observed low efficacy of coupling when mono-oxidized cardiolipin **7** reacted directly with BSA, prothrombin and β2GPI (yields 52–68%). In contrast, using commercially available PEGylated phosphoethanolamine-azide, antigens **8, 10** could be prepared in high yields (Fig 1) [9].

In this study, we adjusted conditions for cardiolipin oxidation. We also extended length of a linker for the attachment of cardiolipin to proteins (Fig 1). In addition, phosphoethanolamine modified prothrombin and β2GPI antigens **5**,**6** were prepared (Fig 1). Chemical composition of product antigens was verified by gel electrophoresis and MALDI MS spectra (see S2 Appendix). According to MS data, 10–11 and 12–13 cardiolipin residues were attached to β2GPI and prothrombin, respectively. This is significantly higher than for a previous approach using direct coupling of cardiolipin derivative **7** to proteins (1–2 residues) [9], and might be a result of reduced steric hindrance of the cardiolipin reagent **13** by the extended linker [20].

### Improved binding specificity for new phospholipid-protein conjugates vs. controls

Our next goal was to evaluate the hypothesis that cross-linking biologically complementary phospholipids and proteins in a chemically efficient way would decrease unspecific binding of the resulting antigens. To do this, we carried out series of IgG ELISA assays using human monoclonal antibodies against HIV-1 antigens [14] and polyclonal healthy control sera obtained at Stanford University Hospital (n = 14) and from Immunovision (HNP) [15].

The results of IgG class ELISA for novel antigens and controls obtained using monoclonal antibodies towards HIV-1 antigens and healthy sera samples are shown in Table 1. We observed the highest level of unspecific binding for non-covalent complexes of phospholipids and proteins **1**+**2** and **1**+**3**. In contrast, cross-linked conjugates **8–11** showed only low to no non-specific binding in similar experiments (for example, Table 1, data for **11** compared to **1**+**2** and **1**+**3**). Conjugates **8–10** demonstrated a weak binding to anti-gp41, whereas binding to anti-p24 and B12 antibodies completely disappeared upon conjugation of phospholipid with protein. High reactivity on HIV-1 specific monoclonal antibodies and healthy control sera observed for mixed phospholipid-protein controls could result from the high heterogeneity of non-covalent structures leading to unspecific interactions with polyclonal antibodies. In turn, cross-reactivity of antigens **8**,**10** might be caused by the presence of PEG linker [21]. Alternative linker design for the attachment of phosphoethanolamine to proteins is an objective of our on-going research and will be published in the near future. Finally, the most specific antigens **9**,**11** were further applied in the studies of human samples described below.

**Table 1:** Analysis of binding specificity for cross-linked antigens 8–11 and controls 1–3,12.^a^

Antigen No/details	Binding human monoclonal antibodies:			Response in healthy controls, Stanford University Hospital, HNP (% of samples)
	a-p24	a-gp41	B12	
**8**/PE^PEG^-PT	-	+	-	+/- (12%)
**10**/PE^PEG^-β2GPI	-	+/-	-	+ (6%), +/- (12%)
**9**/CL-PT	-	+/-	-	+/- (6%)
**11**/CL-β2GPI	-	-	-	- (100%)
**2**/β2GPI	-	+/-	-	+/- (25%)
**3**/PT	+/-	-	+/-	+/- (25%)
**1+2**/CL + -β2GPI	+/-	+/-	+/-	+ (44%), +/- (25%)
**1+3**/CL + PT	+/-	+/-	+	+ (25%), +/- (25%)
**2+12**/PE^PEG^+β2GPI	+/-	+/-	+	+ (25%), +/- (12%)
**3+12**/PE^PEG^+β2GPI	+/-	+/-	+	+ (25%), +/- (25%)

^a^ Weak positive (+/-) and positive (+) signal were defined as 2- and 3-fold absorbance signals, respectively, above the mean value for a healthy control group. PE^PEG^, PT, CL, β2GPI are PEGylated phosphoethanolamine, prothrombin, cardiolipin and β2-glycoprotein I, respectively. For chemical structures of antigens and controls, see Fig 1.

### Autoantibody detection in patient samples

The initial ELISA assay was performed manually using microtiter plates coated with antigens **8–11** or control antigens [8,9]. Sample titration experiments showed low response of IgM antibodies to the dilution, whereas IgG antibodies showed reduced signal upon dilution with a high degree of linearity (R^2^ > 0.95; S2 Appendix). Therefore, in this work we focused on the IgG assay. In addition, repeatability of assays, along with stability of new antigens and controls were studied as described before. Our data proves that new antigens have superior levels compared to controls in agreement with previous data (see S2 Appendix) [9].

Next, healthy patients (n = 14) and a cohort of pediatric SLE patients (n = 27), both monitored at Stanford University Hospital, were analyzed. Prior to the studies, total protein concentration in each sample was analyzed by Bradford assay [16]. Having corrected dilution of each sample to the same total protein value, ELISA experiments were performed as we described before [9]. According to our data, binding equilibrium for the autoantibodies in the samples was achieved within 1 h incubation at room temperature (S2 Appendix). Cut-off value for each applied antigen was established as 2- and 3-times mean A_450_ value for healthy patients for the weak positive and positive signal, respectively [17].

Studies of the SLE cohort obtained from Stanford University Hospital under these conditions identified five and seven positive patients (19% and 26%) when antigens **9** and **11** were applied. This was in contrast with nine and three positive patients (33% and 11%) detected using β2GPI and mixed antigen cardiolipin+β2GPI, respectively. Using clinical anti-phospholipid test, 7 positive patients were identified (Table 2).

**Table 2:** Clinical data and results of analyses of the Stanford University Hospital SLE subjects used in this study.*

Clinical parameter:	SLE samples (Stanford University Hospital)					
	9 (+)	9 (-)	11 (+)	11 (-)	aPL + clinical	aPL—clinical
N (subjects)	5	22	7	20	7	20
Female/Male	5/0	18/4	7/0	16/4	7/0	16/4
MDO age (yr) (range)	12 (7–16)	15 (9–17)	12 (10–16)	15 (7–17)	12 (10–16)	15 (7–17)
MSC age (yr) (range)	12 (7–16)	15 (9–19)	12 (10–16)	15 (7–19)	12 (10–16)	15 (7–19)
Median SLE-DAI (range)	10 (6–30)	6 (0–30)	7 (6–30)	6 (0–30)	7 (4–17)	10 (0–30)
Positive Clinical aPL test (%)	40	22	57	15	-	-
ANA positive status (%)	100	100	100	100	100	100
Anti-Smith positive (%)	80	27	57	30	28	40
Renal/total no. samples (%)	60	36	57	35	28	45
CNS/total no. samples (%)	0	0	0	0	0	0
Malar/total no. samples (%)	80	54	71	50	71	50
Lupus anticoagulant/ total no. samples	0	5	0	5	2	3
Mean absolute lymphocyte count (ALC)	5400	1200	4050	1100	2100	1250
Medication	*G1*	*G1*	G2	*G1*	*G1*	*G1*

* MDO = median age at disease onset, MSC = median age at sample collection, PL = phospholipid; Medication group 1 (**G1**): Pred = prednisone, HCQ = hydroxychloroquine, ST = other steroids, N = naproxen, SO = solumedrol, CY = cytoxan; **G2** medication = **G1** –other steroids.

**Fig 2:**
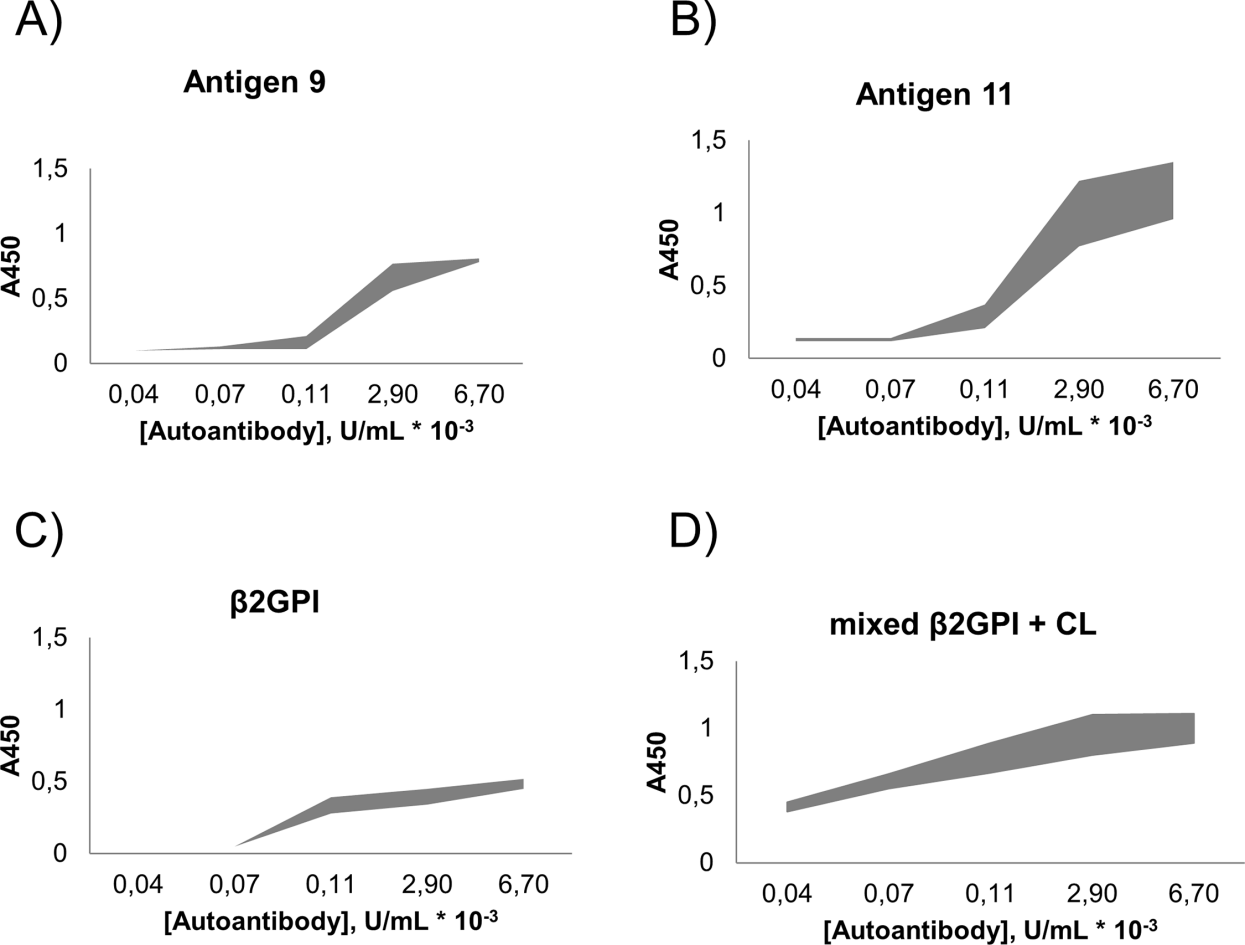
**Dose-response profiles for SLE sera samples, obtained using antigens 9,11 (A-B), β2GPI (C) and mixture of β2GPI with cardiolipin (D).** IgG assay was carried out under the developed indirect ELISA conditions, using randomly selected 16 sera samples (Stanford University SLE cohort), and similar coating and incubation conditions for all antigens. Concentration of autoantibody for the titration in each sample was normalized as a ratio of absorbance using corresponding antigen to total protein in the sample (see Materials and Methods for details).

To get further insight into the nature of detected autoantibodies, their dose-response profiles were obtained (Fig 2) [22]. As can be seen from the resulting profiles, **9**,**11** and control proteins showed different dose response curves for autoantibodies to phospholipid-protein conjugates. Antigens **9** and **11** displayed much sharper saturation curves than β2GPI and its mixture with cardiolipin. Notably, β2GPI showed lowest saturation value among all studied antigens, whereas the highest dispersity among positive samples was observed for antigen **11**. Overall, altered dose-response curves suggested different concentrations and/or autoantibody repertoire detected for antigens **9**,**11** than those for β2GPI and cardiolipin mixture with β2GPI. As a result, we also expected different correlation of the observed autoantibody titers with clinical parameters of the analyzed patients.

**Fig 3:**
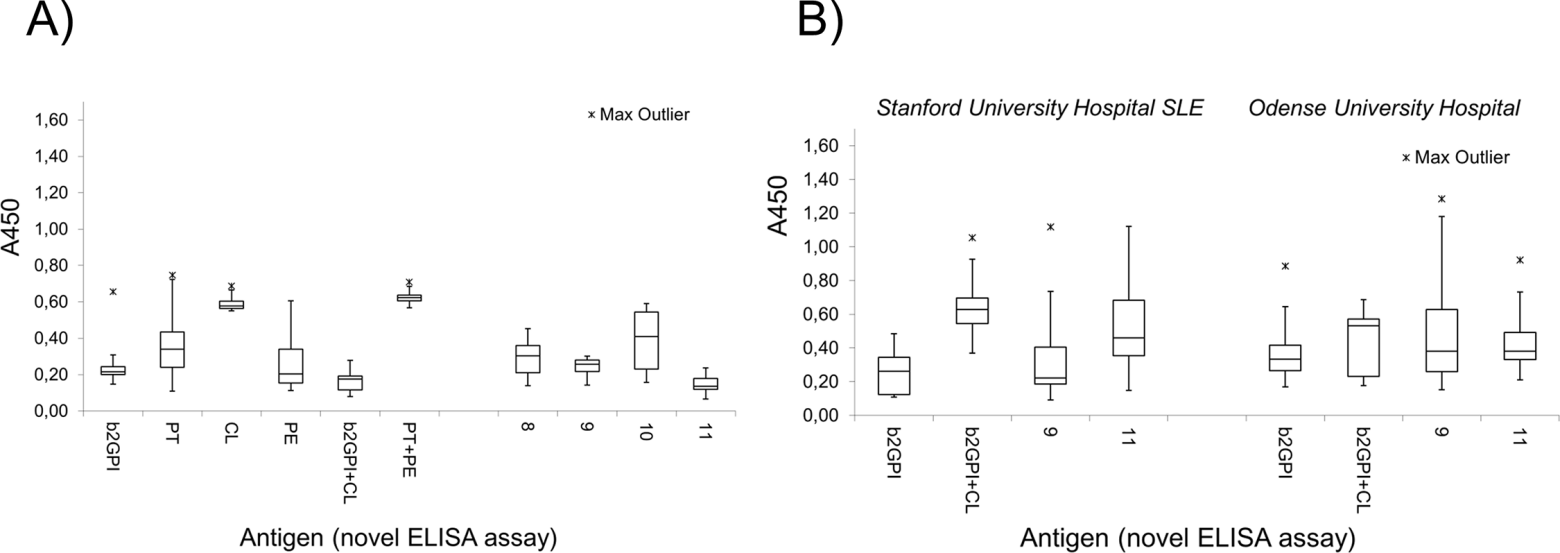
Absorbance values obtained by IgG ELISA assay. The assay was carried out using control antigens, **8–11**, and three cohorts of subjects: adults at Odense University Hospital (n = 34), SLE patients at Stanford University Hospital (n = 27), and healthy controls (Stanford University Hospital; n = 14). CL = cardiolipin; PT = prothrombin; PE = phosphoethanolamine.

Robustness and clinical relevance of the new antigens was further evaluated by the analysis of blood samples from adult patients obtained from Odense University Hospital, Denmark. The assay was performed using similar plate coating, incubation conditions and cut-off values as for SUH cohort, although in a fully automated regimen. The results were compared to commercial anti-cardiolipin and anti-β2GPI tests (Euro Diagnostica), which were performed for Odense University Hospital patients under similar conditions. Summarized ELISA results for healthy controls (Stanford University Hospital) and two patient cohorts (Stanford University Hospital SLE subjects and Odense University Hospital patients) are shown in Fig 3. As can be seen, the background signal of healthy controls was highest for cardiolipin **1**, prothrombin and mixture of phosphoethanolamine with prothrombin. Stanford University Hospital SLE samples showed higher autoantibody titers than the Odense University Hospital cohort, especially when antigen **11** and mixed antigen cardiolipin+β2GPI were applied. Notably, positive samples indicated by **9**,**11** dramatically varied from those indicated by separated antigens. Group analysis using ANOVA confirmed a statistically significant difference between the groups within Stanford University Hospital SLE and Odense University Hospital cohorts with a p value below 0.01 (see Materials and Methods).

Clinical parameters of the studied SLE patients and their autoantibody titers are summarized in Table 2. Notably, similar number of subjects showed presence of antibodies against **9**,**11** compared to clinical test. However, there was no overlap between the patients positive according to clinical anti-phospholipid tests and the developed assay. Next, lupus anticoagulant was positive only for patients with elevated titers of anti-β2GPIs and some patients with clinically determined positivity, but not antibodies binding to **9**,**11**. On the contrary, median value of absolute lymphocyte count (ALC) was elevated for the patients who had autoantibodies binding to **9** and **11**, but not other tested antigens.

**Fig 4:**
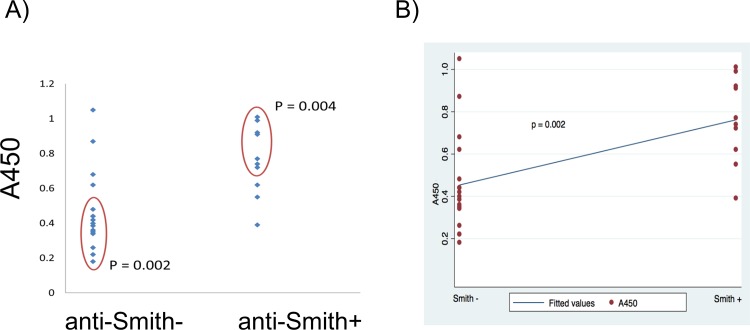
Representative statistical analysis of the data obtained for new antigens. Observed correlations between antibodies to antigen **11** (A450) and anti-Smith results for disease stated patients (Stanford University Hospital SLE cohort): data plot (A) and linear regression plot (B).

Interestingly, 57% and 80% of positive patients detected using phospholipid-protein conjugates were also anti-Smith positive, which is a highly specific antibody for non-drug induced SLE [4,23]. Correlation (p = 0.002) was confirmed by OLS test (Fig 4) [18]. This is in contrast to only 28% and 11% anti-Smith positive samples indicated by clinical test and controls used in this study. Furthermore, 71% and 80% of patients having antibodies to phospholipid-protein conjugates had malar rash and 57–60% were diagnosed with arthritis and SLE nephritis (vs. 15–28%. according to clinical anti-phospholipid test). Overall, median disease activity index (SLE-DAI) [24] was similar for positive samples detected using antigens **9** and **11**. However, antigen **9** detected autoantibodies in patients with slightly more active SLE than **11** (median SLE-DAI 10 vs 7).

Demographic and clinical parameters of Odense University Hospital patients significantly varied from the Stanford University SLE cohort and between each other (Table 3 and S3 Appendix). In general, the former group was not studied in as much detail as the latter. Median age at diagnosis and age at sample was much older for Odense University Hospital cohort (50 vs. 14). Next, no diagnosis was established for 48% of patients, although 90% of them had manifestations of autoimmune conditions and 17 (50%) took no medication prior to sample collection. In Odense University Hospital samples, we observed almost complete overlap of positive results for conjugates **9** and **11** (Table 3). Notably, using antigen **9** in the assay gave lower signal (in a weak positive range only), whereas antigen **11** provided high signal. As in Stanford University Hospital SLE samples, we observed low to no overlap with the results of clinical anti-phospholipid/anti-β2GPI tests.

**Table 3:** Clinical data and results of Odense University Hospital patients showing positivity to phospholipid-protein conjugates.*

Patient nr.	Diagnosis	Manifestations	Binding antigen:				Serology	Medication
			9	11	cardiolipin	β2GPI		
**P3**	Venous thromboembolism, spinal stenosis, hyperlipidemia	Lumbal pain, pulmonary embolism	+	-	-	+	None	Xarelto, ***Atorvastatin***, Gabapentin
**P6**	Hepatocellulary carcinoma	Stomach pain, lumbal pain	+	+++	++	-	None	None
**P8**	Urticarial vasculitis	Skin itch, thrombocytopenia	+	++	-	++	ANA+	Dapson
**P12**	None	Sclerotic aortic valve, TCI symptoms	+	++	-	+	None	Lercanidipin, ***Atorvastatin***,Clopidogrel, Losartan
**P15**	None	None	+	++	++	-	None	None
**P20**	SLE	Arthritis, nephritis, cerebral apoplexia	-	+++	-	++	ANA+, a-dsDNA+	Marevan Plendil ***Simvastatin***
Remai-ning cohort, 28 subjects	SLE (15%), Other (27%), None (48%)	diverse	-	-	+ (22%)	+ (11%)	ANA+ (21%); un-specified	As above (56%); None (44%)

* Drugs potentially causing SLE are indicated in bold italics.

Notably, using separated tests on cardiolipin and β2GPI, either anti-cardiolipin or a-β2GPI positivity has been observed for particular patients. Elevated anti-cardiolipin titers mostly associated with not diagnosed patients who had diverse manifestations of autoimmune conditions (Table 3). On the contrary, high a-β2GPI titers correlated mostly with thrombosis and SLE. Elevated anti-prothrombin titers showed no correlation with any particular disease manifestations (data not shown). In turn, positivity using conjugate **9** correlated with thrombosis and sclerotic manifestations, but not with adult SLE. At last, conjugate **11** provided positivity which associated strongest with renal (kidney) SLE and arthritis.

As a next aspect, we analyzed medical records for Stanford University Hospital SLE and Odense University Hospital patients with respect to medication use. The data were divided into four groups containing one or more medications that are known to induce autoantibodies [25–28], and subjected to statistical analysis. Using OLS tests with and without interaction between variables (eight interactions in total), we confirmed no correlation between a particular medication or a group of medications and elevated titers of autoantibodies to phospholipid-protein conjugates (p > 0.05 with a statistical power 77% for a total of 74 samples).

Finally, we studied repeatability of the developed assay and chemical stability of novel antigens (see Materials and Methods). Our assay was highly reproducible with absorbance values varying within 0.5–0.8 of mean titer for each independent analysis. Moreover, antigens **9**,**11** were stable for up to 3 months at +4°C in glass vials and on the surface of microtiter plates, and up to 1 year in glass vials at -20°C. This is in contrast to cardiolipin which degraded after approx. 3 weeks of storage at +4°C in a glass vial or on the microtiter plate, and after 3 months of storage in a vial at -20°C.

## Discussion

Since 1990s there has been growing evidence on clinical relevance of autoantibodies against plasma proteins binding phospholipids [29–32]. However, role of phospholipids themselves in the autoantibody recognition remained unclear. Separate application of biologically complementary phospholipids and plasma proteins lead to low clinical relevance of detected autoantibodies [30–32]. Moreover, chemical heterogeneity of typically applied antigens extracted from natural sources, their low stability and therefore poor reproducibility of the available assays might contribute to current disagreement between tests [13]. In this study our main goal was to specifically detect clinically important autoantibodies using novel, reliable reagents. We also aimed at contributing to the improved understanding of the correlation between autoantibody titers and the clinical features of a broad range of autoimmune diseases where these antibodies are present.

Synthesis of covalently linked phospholipid-protein conjugates represents a challenging task due to high hydrophobicity of the lipids, their low solubility in aqueous media, instability and low reactivity. Previously, we and others aimed at synthesis of phospholipid-protein conjugates using coupling reactions and click chemistry [9,10]. The latter approach has proved to be more attractive, giving high yields of the phosphoethanolamine derivatives **8**,**10** (Fig 1, 88% and 92%). In this work, we demonstrated that decreasing steric hindrance for cardiolipin using increased linker length provides better coupling yields with protein molecules (**9**,**11**, 89% and 84% vs. **4–6** [9], 54–66%, respectively). Simultaneously, regioselective attachment to proteins by click chemistry could be performed using novel azide **13** (Fig 1).

Having synthesized cardiolipin containing antigens and phosphoethanolamine controls, we evaluated their binding of anti-HIV monoclonal antibodies (B12, a-p21 and a-gp41) and polyclonal antibodies in healthy human controls [29,30]. Previously, we demonstrated high cross-reactivity of anti-phospholipid antibodies on ss and dsDNA [9]. However, this type of cross-reactivity might be caused by shared antigenic determinant for antibodies to phospholipid-protein complexes and aDNAs which is important for their function [3]. Therefore we selected human monoclonal antibodies against different antigens as controls. As can be seen in Table 1, novel antigens showed lower unspecific binding then reference antigens. This is one of the most important results of this paper, because it shows that the binding specificity of phospholipid antigens can be improved by their cross-linking with biologically complementary proteins.

The next important aspect of this work is investigation of autoantibodies in two different cohorts of human patients: children diagnosed with SLE in USA and adults in Denmark (Stanford University Hospital SLE and Odense University Hospital cohorts, respectively). We considered multiple clinical parameters when selecting these patients. Thus, in Stanford University Hospital SLE patients (n = 27), the diagnosis SLE was already established approx. 1 year before sample collection (S3 Appendix). Treatment of these patients included simultaneous application of various medications (see Table 1). In contrast, Odense University Hospital patients were older; 48% of them had no diagnosis and were not treated prior to sampling. The remaining 42% of Odense University Hospital patients had diverse diagnoses and manifestations of autoimmune conditions (Table 3 and S3 Appendix).

IgG class ELISA studies of the patients provided us with the following information on autoantibodies to phospholipid-protein conjugates: 1) binding equilibrium was achieved for all patients within the same incubation time (approx. 1 h, see Materials and Methods); 2) novel antigens showed less non-specific binding and different dose-response curves compared to separated phospholipid and protein antigens; 3) overlapping autoantibody profiles were detected for both cardiolipin-β2GPI and cardiolipin-prothrombin; 4) there is a significant disagreement between currently applied tests and the results provided using new antigens One possible explanation of the different dose-response profiles in patient samples could be different affinity of the detected autoantibodies [22]. We also speculate that antibodies detected by novel antigens could be produced under different clinical circumstances (disease states) than those binding to individual phospholipids and proteins that are used in current tests [8–10,29]. This also could explain different associations between autoantibodies and clinical parameters observed when individual antigen components were applied. Overall, our results experimentally demonstrate statistically significant differences in autoantibody repertoires targeting chemically different antigens.

Previously, antibodies against β2GPI in lupus anticoagulant positive samples were suggested as a biomarker of thrombosis in SLE and APS patients [29]. Indeed, we observed higher lupus anticoagulant activity in patients with positive titers of a-β2GPI when β2GPI is used alone (50%). Most likely due to efficient preventive treatment, thrombolytic events occurred only in 2 (3%) patients used in our study. According to the literature, other a-β2GPI positive patients with lupus anticoagulant positive tests still form a group of risk for thrombosis [29–31]. In turn, positivity using antigen **9** observed in this study (Table 3, patient P3) is a new aspect of predicting thrombosis which might bring additional insights into the disease.

In general, our findings indicate high prognostic and scientific potential of cross-linked phospholipid-protein conjugates. This includes strong correlation of elevated autoantibody titers with high disease activity and in particular with arthritis and renal (kidney) disease, anti-Smith positivity and high absolute lymphocyte count levels. Arthritis and renal disease are common in SLE [33–35]. In turn, anti-Smith antibody is a very specific type of antinuclear antibody which in SLE is associated with central nervous system involvement, kidney disease, lung fibrosis and pericarditis [23,36]. At last, high absolute lymphocyte count (above 5000) is known to be a biomarker of on-going (chronic) inflammation, infections and cancer of the blood or lymphatic system [37]. Recently it was also shown that absolute lymphocyte count values correlates with cancer outcome [37]. Based on this data, positivity using antigens **9**,**11** could contribute positively to diagnosis of high risk profiles in diverse groups of patients having autoimmune diseases.

Subjects selected for this study received medication that could result in a drug-induced SLE and increased titers of autoantibodies (corticosteroids, hydroxychloroquine, cholesterol-lowering atorvastin, simvastin, and combinations thereof; see Tables 2 and 3) [25–29]. Having investigated treatment details for the patients by OLS followed by Holm–Bonferroni correction for multiple comparisons, we concluded that there was no statistically significant correlation between a particular treatment and observed autoantibody titers (adjusted p > 0.05) [19]. The number of data points for this study is relatively small (74 samples were obtained from 61 patients, see S3 Appendix). Nevertheless, our data suggests that with a statistical power of 77% the novel antigens prepared herein allow for the detection of not drug-induced autoantibodies [19].

Finally, according to our studies novel conjugates show higher reproducibility and stability than currently available antigens and have advantage of straightforward synthetic route, high chemical homogeneity and decreased cross-reactivity on nonspecific human antibodies (Table 1; Materials and Methods).

In conclusion, this work describes a robust synthetic route leading to efficient phospholipid-protein antigens for the detection of human autoimmune antibodies. To achieve necessary specificity and sensitivity of autoantibody detection, we cross-linked biologically complementary phospholipids and proteins under mild, effective conditions. In doing this, we found it necessary to extend the linker between sterically hindered cardiolipin and proteins for further conjugation by click chemistry. This approach is not limited to cardiolipin-β2GPI and cardiolipin-prothrombin conjugates, but can be potentially expanded to other clinically relevant antigens, such as derivatives of sphingomyelin, ethanolamine etc. [38–40] As we prove in this work, the cross-linked phospholipid-protein conjugates have advantages of high specificity towards autoantibodies in human samples. Moreover, detected autoantibodies may also play a role in pathogenesis of various autoimmune diseases. Successfully demonstrated as a proof-of-principle in two cohorts of patients diagnosed or having manifestations of autoimmune diseases (Stanford University Hospital SLE cohort, n = 27, and Odense University Hospital patients, n = 34), this method can be applied to detect antibodies to phospholipid-protein complexes in other autoimmune conditions. In particular, these autoantibodies are of high relevance in neurological autoimmunity, e.g. autoimmune demyelination conditions leading to multiple sclerosis and other diseases [41,42]. Reliable synthetic antigens could yield new insights into the pathogenesis of these diseases and simultaneously serve as a simple, reliable method for research, diagnosis, prognosis and point-of-care monitoring of patients.

## Supporting Information

S1 Appendix List of abbreviations. (PDF)

S2 Appendix Synthesis and characterization of protein-phospholipid antigens. Preparation of azide **13** and its conjugation to proteins is described. Products are characterized by gel electrophoresis, mass spectrometry and ELISA with polyclonal plasma controls.
(PDF)

S3 Appendix Subjects demographics and clinical parameters at individual level and in general. Included parameters: age at disease onset and age at sample, gender, ethnicity, laboratory diagnostics, medication.
(PDF)

S4 Appendix Individual-level data used for Figs 2–4. (PDF)
