# Supplementary material for: Novel Phospholipid-Protein Conjugates Allow Improved Detection of Antibodies in Patients with Autoimmune Diseases
Source: PLoS One. 2016 Jun 3;11(6):e0156125. doi: 10.1371/journal.pone.0156125 (PMC4892602; doi:10.1371/journal.pone.0156125)
Supplement: S1 Appendix — (PDF) [file pone.0156125.s001.pdf]

S1 Appendix. List of Abbreviations

APS	Antiphospholipid antibody syndrome
PLPs	Phospholipid-protein complexes
SLE	Systemic lupus erythematosus
β_2GPI	β_2-glycoprotein I
PT	Prothrombin
CuAAC	Copper-catalyzed azide-alkyne cycloaddition
ELISA	Enzyme-linked immunosorbent assay
STP	Pentynoic sulfotetrafluorophenyl
AIDS	Acquired immune deficiency syndrome
NIH	National Institutes of Health
HRP	Horseradish Peroxidase
HNP	Herniated nucleus pulposus
OLS	ordinary least squares analysis
BSA	Bovine serum albumin
PE^{PEG}	PEGylated phosphoethanolamine
CL	Cardiolipin
I_gM	Immunoglobulin M
I_gG	Immunoglobulin G
LA	lupus anticoagulant
ALC	Absolute lymphocyte count
DAI	disease activity index
MDO	median age at disease onset
MSC	median age at sample collection
Pred	prednisone
HCQ	hydroxychloroquine

ST	other steroids
N	naproxen
SO	solumedrol
CY	cytoxan
ANA	anti-nuclear antibody
a-dsDNA	antibodies towards double stand deoxy ribonucleic acid
