# Supplementary material for: Novel Phospholipid-Protein Conjugates Allow Improved Detection of Antibodies in Patients with Autoimmune Diseases
Source: PLoS One. 2016 Jun 3;11(6):e0156125. doi: 10.1371/journal.pone.0156125 (PMC4892602; doi:10.1371/journal.pone.0156125)
Supplement: S2 Appendix — Preparation of azide 13 and its conjugation to proteins is described. Products are characterized by gel electrophoresis, mass spectrometry and ELISA with polyclonal plasma controls. (PDF) [file pone.0156125.s002.pdf]

# S2 Appendix. Synthesis and characterization of protein-phospholipid antigens

## Synthesis of new antigens

In general, using microwave promoted oxidation of cardiolipin and reaction with *N*-hydroxysuccinimide ester/*N,N′*-diizopropylcarbodiimide we were able to obtain compound **16,** which was afterwards reacted with 3-azidopropan-1-amine (Scheme S1). The product azide **13** was successfully applied in the CuAAC click chemistry with protein-alkyne derivatives **14-15** under similar conditions as described before.[1]

**Scheme S1.** Synthesis strategies for cardiolipin-β2GPI and cardiolipin-prothrombin complexes.

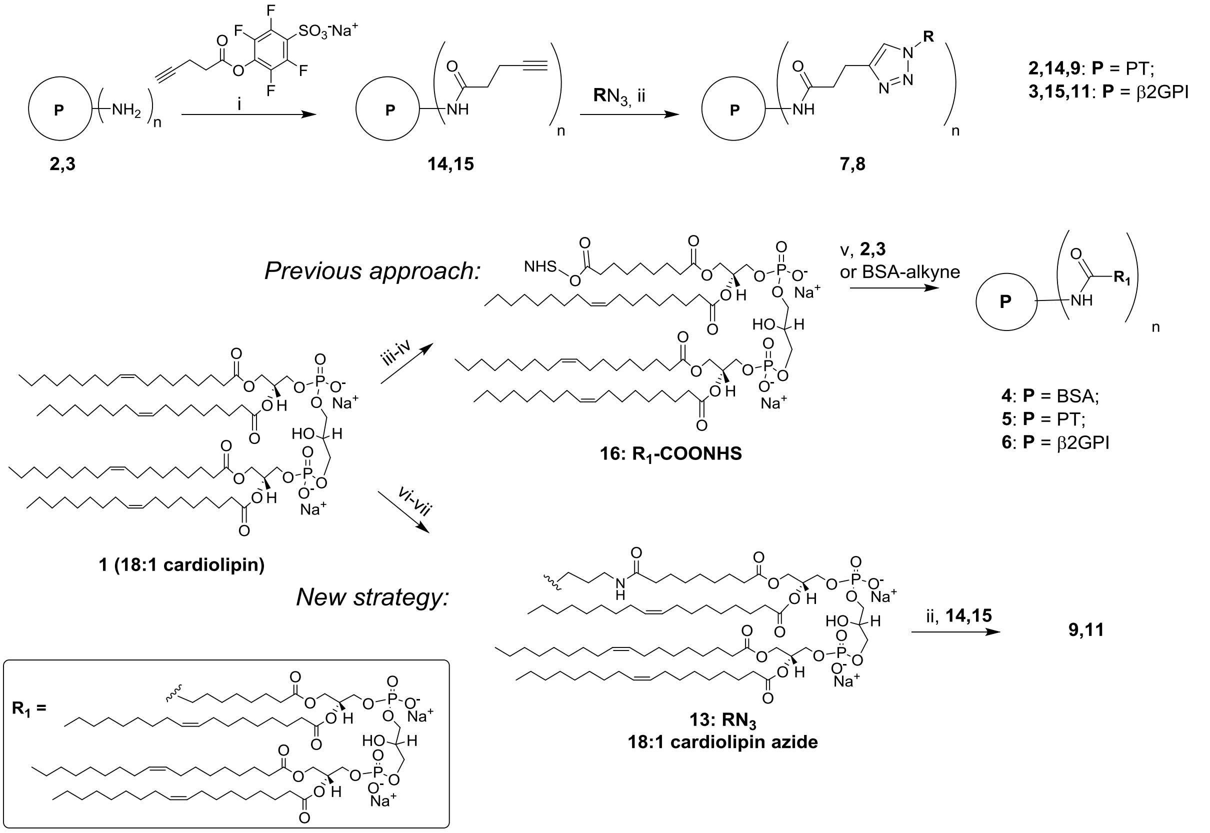


*Reagents and conditions*: (i) 0.1 M bicarbonate buffer–DMSO 9:1, 4°C, 12 h; (ii) corresponding azide, $CuSO_4$:TBTA 1:1.1, ascorbic acid, 1× PBS-DMSO–*t*-BuOH 3:2:0.1, v/v/v; (iii) $KMnO_4$, $NaIO_4$, *t*-BuOH–$H_2O$ 9:1, v/v, rt, 12 h; (iv) succinimide ester, *N,N′*-diisopropylcarbodiimide, DMSO, rt, 12 h; (v) proteins **2**,**3**, 0.1 M bicarbonate buffer–DMSO 9:1, v/v, rt, 12 h; (vi) $KMnO_4$, $NaIO_4$, *t*-BuOH–$H_2O$ 9:1, v/v, 45 °C, 1 h, microwave reactor; (vii) succinimide ester, *N,N′*-diisopropylcarbodiimide, DMSO, rt, 5 h; → 3-azidopropan-1-amine, rt, 12 h.

[1] See Maity A, Macaubas C, Mellins E, Astakhova K. Synthesis of Phospholipid-Protein Conjugates as New Antigens for Autoimmune Antibodies. Molecules. 2015;20(6):10253-10263.

**Preparation of azide 13**

A solution of cardiolipin (CL) **1** (10 mg, 6.66 µmol) in 1.5 mL tBuOH 100 µL was mixed with $NaHCO_3$ (10 mg in 100 µL), $NaIO_4$ (30 mg in 200 µL water) and $KMnO_4$ (10 mg in 200 µL water) [17]. The reaction was flashed with argon for 3 min and afterwards kept in a microwave reactor (15V) for 1 h at 45 °C. After the starting material was no longer detectable by TLC, the reaction was quenched by adding 150 mg $Na_2SO_3$. The mixture was afterwards acidified with 5% HCl to pH 3.0 and washed twice with *t*-BuOH. *t*-BuOH fraction was dried over $Na_2SO_4$ and evaporated *in vacuo*. Yield 86%; $R_f$ 0.37 (chloroform:methanol:water 3:1.5:0.2, v/v/v), HRMS-ESI *m*/*z*: 1406.84772 ($[M + Na]^+$, $C_{72}H_{130}Na_2O_{19}P_2$ calcd 1406.84766).

To a solution of oxidized cardiolipin (5 mg) in 1 mL DMSO 4 mg succinimide ester in 20 µL DMSO and 8 µL *N,N′*-diisopropylcarbodiimide were added. After keeping the reaction for 5 h at room temperature TLC showed complete conversion of the starting material. Intermediate NHS-cardiolipin was further reacted with 3-azidopropan-1-amine (1.1 eq., 0.35 mg) at room temperature in dark over 12 h. The product was used in further steps without purification. $R_f$ 0.55 (chloroform:methanol:water 3:0.5:0.2, v/v/v), HRMS-ESI *m*/*z*: 1488.91209 ($[M + Na]^+$, $C_{75}H_{136}N_4Na_2O_{18}P_2$ calcd 1488.91203).

**Click reactions** were performed following a previously described procedure [11]. The products were initially purified by gel filtration using Zeba spin desalting columns (Life Technologies), and afterwards precipitated from cold acetone (-20 °C). The resulting conjugates were washed twice with cold acetone, dried *in vacuo* and analyzed by mass spectrometry and gel electrophoresis (Supporting Information). Final yields of products based on the absorbance at 280 nm: 88% (**8**), 92% (**10**), 89% (**9**), 84% (**11**).

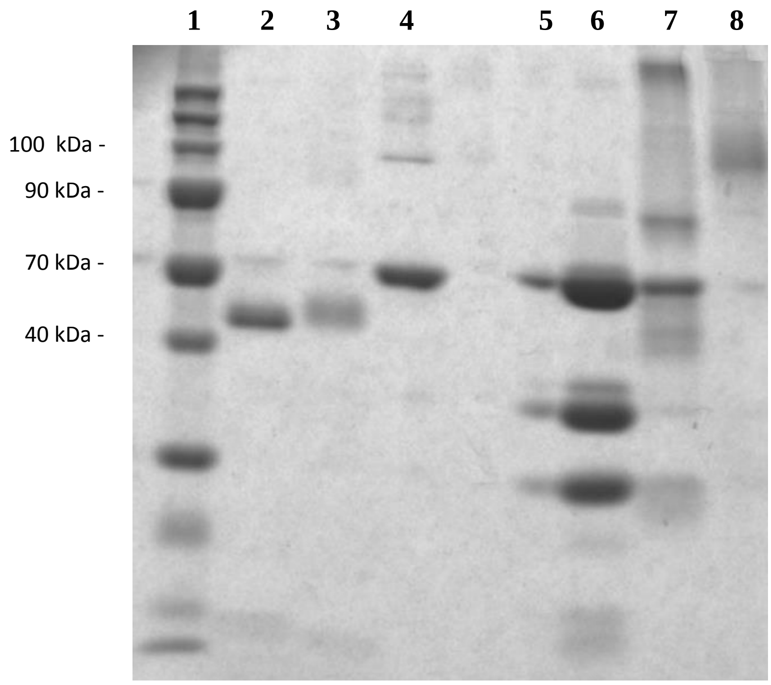


**Fig S1. SDS-PAGE gel electrophoresis of starting proteins.** Loaded samples: ladder (lane 1), β2GPI (lane 2), PT 0.6 ng, 2 ng, lanes 5-6, and product conjugates **6** (lane 3), **11** (lane 4), **5** (lane 7), **9** (lane 8). The 12% SDS-PAGE gel was run at 100 V in 1xTris buffer (pH 7.4), and stained using Coomassie blue overnight.

Prothrombin gives three bands on denaturing SDS-PAGE in agreement with previous reports.[2]

[2] Wang QQ *et al* / Acta Pharmacol Sin 2004 Apr; 25 (4): 514-521.

**Table S1.** MALDI MS results for unmodified proteins and conjugates prepared in this study.[3]

| Protein/Con-jugate | Applied lipid reagent | MS calcd, kDa | MS found, kDa | Number of modifications, n |
|---|---|---|---|---|
| **prothrombin** | - | 72.0 | 72.0 | - |
| **5** | **13** (oxidized CL) | 73.6-75.1 | 73.2-75.4 | 1-2 |
| **9** | **10** | 91.4-93.0 | 91.0-93.4 | 12-13 |
| **β2GPI** | - | 50.0 | 50.0 | - |
| **6** | **13** (oxidized CL) | 53.1 | 52.8 | 2 |
| **11** | **10** | 66.2-67.8 | 66.0-68.1 | 10-11 |

[3] For representative MALDI-MS spectra see: Maity A, Macaubas C, Mellins E, Astakhova K. Synthesis of Phospholipid-Protein Conjugates as New Antigens for Autoimmune Antibodies. Molecules. 2015;20(6):10253-10263.

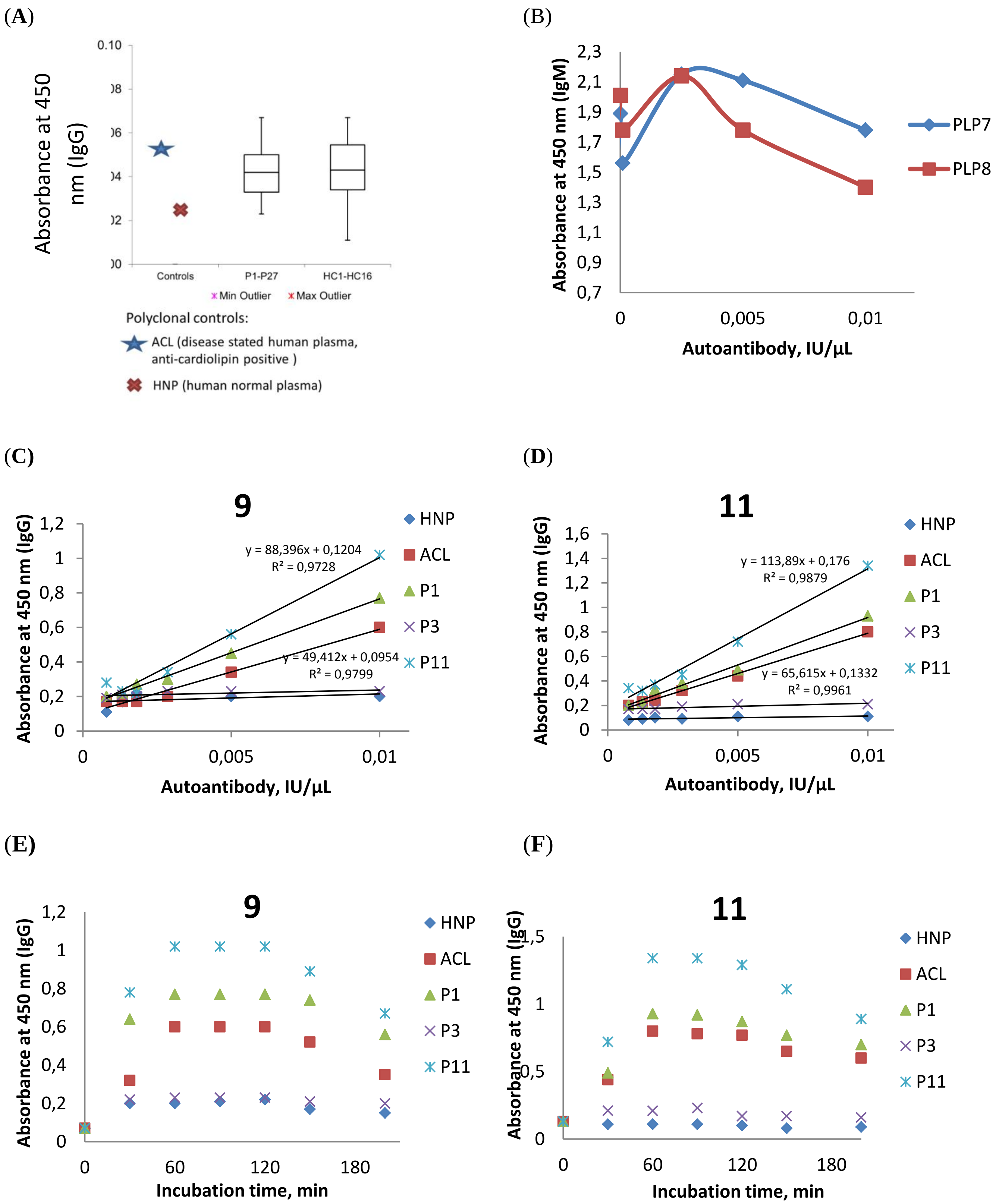


**Fig S2. Characterization of aPLP IgG ELISA assay developed in this study.** A) Boxplot signal of plasma samples P1-P27 and healthy controls (HC1-HC16) on empty plate; plasma dilution 1:100; B) IgM titration of ACL control on PLP7-8 coated plates; C-D) plasma titration (ACL, HNP, P1, P5, P11 (Stanford University Hospital SLE cohort)) using antigens **9** and **11**; E-F) Representative determination of equilibrium time for aPLP biniding (Stanford University Hospital SLE patients): absorbances over time course for primary incubation are presented); antigens **9** and **11**, plasma dilutions 1:100.
