# Supplementary material for: Novel Phospholipid-Protein Conjugates Allow Improved Detection of Antibodies in Patients with Autoimmune Diseases
Source: PLoS One. 2016 Jun 3;11(6):e0156125. doi: 10.1371/journal.pone.0156125 (PMC4892602; doi:10.1371/journal.pone.0156125)
Supplement: S3 Appendix — Included parameters: age at disease onset and age at sample, gender, ethnicity, laboratory diagnostics, medication. (PDF) [file pone.0156125.s003.pdf]

S3 Appendix. Patient demographics and clinical data

Table S1. Stanford University Hospital healthy controls: demographics in general

Variable	Subjects
N (subjects)	14
Female/Male	5/9
White	8
Black	0
Asian	4
non-Hispanic	6
Hispanic	2
Ethnicity unknown	2
Median age (yr) at sample collection (range)	23.3 (3.6-24.7)

Table S2. Stanford University Hospital healthy controls: demographics of individual subjects

Control ID	Gender	Age at sample	Race	Ethnicity
142A	M	8.6	White	non-Hispanic
169A	F	7.9	White	Hispanic
171A	M	3.6	Asian	non-Hispanic
304A	F	24.7	White	non-Hispanic
305A	F	23.7	Asian	non-Hispanic
306A	F	23.4	Asian	non-Hispanic
307A	M	23.4	White	Hispanic
310A	M	24.3	Asian	non-Hispanic
311A	M	24.3	White	non-Hispanic
312A	M	23.2	White	non-Hispanic
313A	F	23.3	unknown	unknown
314A	M	23.2	unknown	unknown
324A	M	13.5	White	non-Hispanic
393A	M	16.1	White	non-Hispanic

Table S3. Stanford University Hospital SLE cohort: demographics in general.[a]

**Variable**	**Subjects**
Number (%) females	24 (89)
White	20
Black	0
Asian	4
Hawaiian/Pacific Islander	1
Race unknown	2
non-Hispanic	6
Hispanic	16
Age at diagnosis, median (range) years	14.1 (7.5-17.6)
Age at sample collection, median, (range) years	14.5 (7.5-19.2)
dsDNA antibody positive patients (%)	19 (70)
Class III/IV lupus nephritis-biopsy proven† (%)	10 (37)
Mean (range) SLEDAI score	11.6 (4-30)
Mean (range) ESR	48 (1-132)
Mean (range) C3	72 (24-121)
Treatment at sample:	
PO steroids (%)	13 (48)
IV steroids (%)	7 (26)
HCQ (%)	17 (63)
Other IS (%)	4 (15)
Daily NSAID (%)	6 (22)
/Other/None	2 (7)

[a] HCQ = hydroxychloroquine, IS = immunosuppressive therapy (cyclophosphamide, mycophenolate, or methotrexate).

Table S4. Stanford University Hospital SLE cohort: demographics and clinical data of individual subjects

Sample nr.	Gender	Age Diag(yrs)	Age sample(yrs)	SLEDAI	Ethnicity	Race	8+	10+	b2GPI+	CL+	Plat	ESR	C3	C4	DNA Ab
5A	F	13.9	14.0	8	non-Hispanic	White	neg	neg	neg	neg	276	6	98	14.7	1:40
5B	F	13.9	14.4	6			neg	neg	neg	neg	402	3	107	nd	neg
5D	F	13.9	15.0	0			neg	neg	neg	neg	401	7	120	23.3	neg
24F	F	15.7	17.3	4	Hispanic	White	neg	neg	pos	neg	228	n.d.	83	12.9	nd
29A	F	10.0	10.1	6	Hispanic	White	pos	pos	neg	neg	262	21	82	3	neg
29C	F	10.0	10.7	2			pos	pos	neg	neg	301	15	100	6.5	neg
29E	F	10.0	11.1	2			pos	pos	neg	neg	174	15	99	9	neg
31A	F	11.5	11.5	4	non-Hispanic	White	neg	neg	pos	neg	246	40	86	6.4	1:80
31V	F	11.5	16.8	0			neg	neg	pos	neg	245	9	132	22.6	neg
40A	F	14.1	14.1	17	Hispanic	White	neg	neg	pos	neg	260	50	33	2.3	1:80
43AA	F	12.7	19.2	16	Hispanic	White	neg	neg	neg	neg	330	45	121	11.8	nd
49A	F	16.2	16.2	7	Hispanic	Unknown	pos	pos	pos	neg	37	110	71	5.1	neg
51A	F	15.3	15.4	10	Hispanic	White	neg	neg	neg	neg	305	65	42	3.1	1:5120
52A	F	13.7	13.8	6	Hispanic	Unknown	neg	pos	neg	neg	221	3	84	nd	neg
56A	F	17.5	17.6	12	Hispanic	White	neg	neg	neg	pos	272	122	64	5	1:5120
56B	F	17.5	17.9	8			neg	neg	neg	neg	257	31	100	23.6	1:40
60D	F	15.7	16.4	8	non-Hispanic	White	neg	neg	neg	neg	308	30	87	15.4	1:10240
61A	M	15.0	15.0	27	Hispanic	White	neg	neg	neg	neg	141	99	26	2.6	1:20480
61B	M	15.0	15.2	6			neg	neg	neg	neg	196	nd	85	17.8	neg
61D	M	15.0	15.6	4			neg	neg	neg	neg	213	0	115	21.5	neg
67A	F	12.0	12.1	6	Hispanic	White	neg	pos	pos	neg	378	0	62	6.7	1:80
69A	M	13.2	13.2	25	non-Hispanic	Asian	neg	neg	pos	neg	100	80	18	2.7	1:5120
69B	M		13.5	4			neg	neg	neg	neg	297	40	62	8.7	1:640
69D	M	13.2	14.1	18			neg	neg	neg	neg	244	42	35	3.6	1:10240
69I	M		15.1	6			neg	neg	neg	neg	250	14	104	15	neg
70A	F	16.3	16.3	30	non-Hispanic	Pacific Islander	pos	pos	neg	pos	194	132	25	<5	1:81920
70B	F		16.6	13			neg	neg	neg	neg	95	30	85	21.8	neg
70E	F		17.3	8			neg	neg	neg	neg	315	35	140	43.7	neg
71A	F	14.5	14.5	30	non-Hispanic	Asian	neg	neg	neg	neg	139	119	28	2.2	1:5120
72A	F	7.5	7.5	10	Hispanic	White	pos	neg	neg	neg	223	55	87	9.5	neg
74B	F	9.5	9.8	6	Hispanic	White	neg	neg	neg	neg	408	12	111	12.6	1:20
75C	F	9.6	10.2	2	non-Hispanic	White	neg	neg	neg	neg	369	7	81	13	1:80
77D	F	16.6	17.3	14	Hispanic	White	neg	neg	neg	neg	295	12	120	22.8	neg
78A	F	17.0	17.0	5	Hispanic	White	neg	neg	pos	neg	399	15	86	4.1	1:80
80A	F	10.9	11.0	4	non-Hispanic	White	neg	neg	neg	neg	324	1	95	7.5	1:40
81A	F	12.2	12.3	26	Hispanic	White	pos	pos	neg	neg	140	109	24	2	1:20480
82A	F	16.4	16.4	6	non-Hispanic	White	neg	neg	neg	neg	361	47	91	17	neg
85A	F	16.2	16.3	4	non-Hispanic	Asian	neg	neg	pos	neg	196	31	115	32.2	neg

96A	F	11.8	11.8	17	non-Hispanic	Asian	neg	pos	neg	pos	224	6	33	5.9	>1:5120
98A	M	17.6	17.6	4	Hispanic	White	neg	neg	neg	neg	281	38	84	10.3	neg

Sample nr.	G/C Casts	U-P:C ratio	Smith	RNP	Ro	La	APL	ANCA	Nephritis	Renal
5A	no	0.11	neg	neg	neg	neg	Pos	nd	No	No
5B	no	0.09	nd	nd	nd	nd	nd	nd	No	No
5D	no	0.07	nd	nd	nd	nd	neg AC	nd	No	nd
24F	no	0.19	nd	nd	nd	nd	nd	nd	No	Yes
29A	no	0.19	neg	neg	neg	neg	Pos	Pos	Yes	Yes
29C	no	0.11	nd	nd	nd	nd	nd	nd		
29E	no	0.05	nd	nd	nd	nd	Pos	nd	nd	Yes
31A	no	0.15	neg	neg	neg	neg	Pos	Pos	No	nd
31V	no	0.21	nd	nd	nd	nd	nd	nd	No	nd
40A	Yes	2.35	neg	neg	neg	neg	neg	neg	Yes	Yes
43AA	no	0.27	nd	nd	nd	nd	nd	nd		
49A	no	0.09	Pos	neg	neg	neg	Pos	neg	No	No
51A	no	0.15	Pos	Pos	neg	neg	Pos	nd	No	No
52A	no	0.06	Pos	Pos	neg	neg	neg	nd	No	No
56A	no	0.20	neg	neg	neg	neg	neg	nd	Yes	Yes
56B	no	0.08	nd	nd	nd	nd	nd	nd		
60D	no	0.13	nd	nd	nd	nd	nd	nd	No	No
61A	Yes	1.46	Pos	Pos	neg	neg	neg	neg	Yes	Pos
61B	no	0.32	nd	nd	nd	nd	nd	nd		
61D	no	0.07	nd	nd	nd	nd	nd	nd	No	Yes
67A	no	0.41	neg	neg	Pos	Pos	Pos	neg	No	No
69A	no	0.67	Pos	Pos	Pos	Pos	neg	neg	No	No
69B	no	0.11	nd	nd	nd	nd	nd	nd	No	No
69D	no	0.19	nd	nd	nd	nd	nd	nd	Yes	Yes
69I	no	0.20	nd	nd	nd	nd	neg	nd		
70A	Yes	0.64	Pos	Pos	neg	neg	neg	neg	Yes	Yes
70B	Yes	17.61	nd	nd	nd	nd	nd	nd		
70E	no	2.83	nd	nd	nd	nd	nd	nd		
71A	no	0.57	neg	neg	Pos	Pos	neg	nd	Yes	Yes
72A	no	0.15	Pos	Pos	neg	neg	neg	neg	No	No
74B	no	0.2	nd	nd	nd	nd	nd	nd	No	No
75C	no	0.14	nd	nd	nd	nd	nd	nd	Yes	Yes
77D	Yes	4.86	nd	nd	nd	nd	nd	nd	Yes	Yes
78A	no	0.21	neg	Pos	neg	neg	neg	nd	No	nd
80A	no	0.34	Pos	Pos	neg	neg	neg	nd	Yes	Yes
81A	Yes	1.45	Pos	neg	Pos	neg	neg	neg	Yes	Yes

82A	no	0.14	neg	neg	neg	neg	neg	nd	No	nd
85A	no	0.13	neg	neg	Pos	Pos	neg	neg	No	nd
96A	no	1	neg	Pos	Pos	Pos	Pos	nd	Yes	Yes
98A	no	0.1	Pos	Pos	neg	neg	neg	nd	No	nd

**Sample nr.**	**Malar**	**Discoid**	**Photosens**	**Oral Ulcers**	**Arthritis**	**Serositis**	**Renal**	**CNS**	**Heme**	**AutoAb**	**ANA**
5A	Yes	No	No	No	Yes	No	No	No	Yes	Yes	1:1280
5B											nd
5D											nd
24F											1:640
29A	No	No	No	No	Yes	No	Yes	No	No	Yes	1:640
29C											1:640 (5/11/04)
29E											nd
31A	Yes	No	No	Yes	Yes	No	No	No	Yes	Yes	1:1280
31V											nd
40A	No	No	No	No	No	No	Yes	No	Yes	Yes	1:320
43AA	No	No	No	No	Yes	No	Yes	No	No	Yes	nd
49A	No	No	No	No	Yes	No	No	No	Yes	Yes	1:640
51A	Yes	No	Yes	Yes	No	No	No	No	Yes	Yes	1:1280
52A	Yes	Yes	Yes	No	No	No	No	No	Yes	Yes	1:320
56A	No	No	No	No	Yes	No	Yes	No	Yes	Yes	1:1280
56B											1:1280
60D	Yes	No	No	No	Yes	Yes	No	No	No	Yes	nd
61A	No	No	No	Yes	Yes	No	Yes	No	Yes	Yes	1:2560
61B											1:40
61D											nd
67A	Yes	No	No	No	Yes	No	No	No	No	Yes	1:2560
69A	Yes	No	No	Yes	No	No	No	No	Yes	Yes	1:1280
69B											nd
69D							Yes				1:1280
69I											nd
70A	Yes	No	Yes	No	Yes	No	Yes	No	Yes	Yes	1:2560
70B											nd
70E											nd
71A	Yes	No	No	Yes	Yes	Yes	Yes	No	Yes	Yes	1:1280
72A	Yes	No	No	Yes	Yes	No	No	No	Yes	Yes	1:2560
74B	No	No	No	Yes	Yes	No	No	No	Yes	Yes	nd
75C	Yes	Yes	Yes	No	No	No	Yes	No	Yes	Yes	nd
77D	Yes	No	No	No	Yes	Yes	Yes	No	Yes	Yes	nd

78A	No	No	No	Yes	Yes	Yes	No	No	Yes	Yes	1:1280
80A	Yes	No	Yes	No	Yes	No	Yes	No	Yes	Yes	1:1280
81A	Yes	No	No	Yes	Yes	No	Yes	No	Yes	Yes	1:2560
82A	No	No	No	No	Yes	No	No	No	Yes	Yes	1:80
85A	Yes	No	Yes	Yes	No	No	No	No	Yes	No	1:640
96A	Yes	No	No	No	Yes	No	Yes	No	Yes	Yes	>1:2560
98A	Yes	No	No	No	Yes	No	No	No	Yes	Yes	>1:2560

**Sample nr.**	**Exam/Symptoms**	**SLE Treatment at time of sample**
5A	arthritis, rash	Prednisone 20 mg BID, HCQ 200 mg daily, Vioxx 25 mg daily, ranitidine
5B	malar rash, hair loss	Prednisone 10 mg daily, HCQ 300 mg daily, Vioxx 25 mg AM, 12.5 mg PM, baby aspirin, ranitidine
5D	normal	Prednisone 7.5 mg daily, HCQ 300 mg daily, indomethacin 25 mg daily, baby aspirine, ranitidine
24F	arthritis in knees	Prednisone 10 mg daily, HCQ 300 mg daily, baby aspirin
29A	No complaints	Prednisone 60 mg daily, HCQ 200 mg daily, ranitidine
29C	no complaints	Pred 7.5 mg daily, HCQ 200 mg daily, baby aspirin, received Solumedrol & CTX this visit
29E	no complaints	Pred 7.5 mg daily, HCQ 200 mg daily, baby aspirin
31A	malar rash, joint pain	Ibuprofen as needed
31V	Migraines	Plaquenil 400 mg daily, baby aspirin, Topamax
40A	leg pain, edema	IV Solumedrol 1000 mg 3/17/2004 & 3/19/2004, antihypertensive therapy
43AA	arthritis, rash, ulcer	Prednisone 5 mg daily, HCQ 300 mg daily, baby aspirin, methimazole, propranolol
49A	arthritis, fatigue	IV Solumedrol 1000 mg 7/31/04, 8/1/2004, 8/2/2004 then Prednisone 30 mg BID, HCQ 400 mg daily
51A	malar rash, ulcers	Prednisone 50 mg daily, HCQ 400 mg daily, ranitidine
52A	malar rash	HCQ 200 mg daily, tacrolimus ointment
56A	arthritis, fatigue	Prednisone 30 mg BID, ranitidine
56B	hair loss	none (self discontinued medications)
60D	joint pain	Prednisone 10 mg daily, HCQ 300 mg daily, naproxen, ranitidine
61A	arthritis, ulcers	IV Solumedrol 1000 mg 12/21-12/23/2005
61B	No complaints	Prednisone 30 mg BID, HCQ 400 mg daily, ranitidine, IV Cytoxan & Solumedrol this visit
61D	knee arthritis	Prednisone 7.5 mg daily, HCQ 200 mg daily (CellCept started at this visit, was not on at time of sample)
67A	hair loss, rash	Prednisone 50 mg daily, HCQ 200 mg daily, baby aspirin, ranitidine (unclear why off naproxen)
69A	oral ulcers, rash	IV Solumedrol 1000 mg 3/28-3/30/2006, (discharged home 3/30 on Pred 50 mg BID, HCQ 300 mg daily
69B	No complaints	Prednisone 7.5 mg daily, HCQ 300 mg daily
69D	Malar rash, vasculitis	Prednisone 7.5 mg daily, HCQ 300 mg daily, methotrexate 20 mg weekly (PO)
69I	No complaints	Prednisone 7.5 mg daily, HCQ 300 mg daily, CellCept 1000 mg BID
70A	Arthritis, rash	IV Solumedrol 1000 mg 4/20-4/22/06, received first Cytoxan 4/26/06
70B	Edema, cough	Prednisone 40 mg BID, HCQ 400 mg daily, omeprazole, multiple renal meds. Received IV Solumedrol & Rituximab this visit
70E	Loose stools	Prednisone 5 mg daily, CellCept 250 mg BID, Lovenox, Prilosec, renal meds
71A	Arthritis, ulcers	Sample obtained before treatment
72A	Malar rash, arthritis	HCQ 100 mg daily, Naproxen
74B	Malar rash	Prednisone 20 mg AM, 15 mg PM, HCQ 200 mg daily, naproxen, methotrexate 12.5 mg weekly (PO)
75C	Headaches, hair loss	Prednisone 5 mg daily, HCQ 100 mg daily, ranitidine. Received 6th IV Solumedrol & Cytoxan this visit

77D	Malar rash, cough	Prednisone 10 mg daily, CellCept 1000 mg BID, HCQ 400 mg daily, baby aspirin
78A	Arthritis	Prednisone 60 mg daily, HCQ 400 mg daily, baby aspirin, ranitidine
80A	No complaints	Prednisone 30 mg BID, CellCept 750 mg AM/500 mg PM, HCQ 150 mg daily, ranitidine
81A	Malar rash, fever	received 3 IV solumedrol pulses 10/23-10/25
82A	Arthritis	Piroxicam 10 mg daily
85A	Malar rash, ulcer	HCQ 200 mg daily. Started IV Solumedrol pulses this visit (after sample obtained)
96A	Arthritis, malar rash	On IV Solumedrol 96 mg BID when sample obtained
98A	Malar rash, arthritis	Naproxen 500 mg BID, ranitidine

Table S5. Odense University Hospital cohort: demographics and clinical data in general

Variable	Subjects
N (subjects)	34
Female/Male	14/13
Gender unknown	7
White	29
Black	0
Asian	5
non-Hispanic	31
Hispanic	0
Median age (yr) at sample collection (range)	50 (18-85)
Diagnosis: SLE/Other/None	5/11/18
Clinical anti-cardiolipin IgG positive (% subjects)	2 (6)
Clinical anti-b2GPI IgG positive (% subjects)	18 (53)
Medication: Statins/Other/None	17/4/17

Table S6. Odense University Hospital SLE cohort: demographics and clinical data of individual subjects

Patient nr	Gender	Age (sample)	Race	Ethnicity	Clinical test a-CL (IgG)	Clinical test a-b2GPI (IgG)	Novel assay 9+	11+
P1	F	45	White	non-Hispanic	neg	neg	neg	neg
P2	M	79	Asian	non-Hispanic	neg	Pos	neg	neg
P3	nd	nd	White	non-Hispanic	elevated	elevated	Pos	neg
P4	M	50	White	non-Hispanic	neg	Pos	neg	neg
P5	M	59	White	non-Hispanic	neg	neg	neg	neg
P6	nd	nd	White	non-Hispanic	neg	neg	neg	elevated
P7	M	23	White	non-Hispanic	neg	Pos	neg	neg
P8	nd	nd	White	non-Hispanic	neg	neg	Pos	Pos
P9	M	50	White	non-Hispanic	neg	Pos	neg	neg
P10	F	76	White	non-Hispanic	neg	elevated	neg	neg
P11	M	65	White	non-Hispanic	neg	Pos	neg	neg
P12	nd	nd	White	non-Hispanic	neg	Pos	Pos	Pos
P13	F	47	White	non-Hispanic	Pos	neg	neg	neg
P14	F	18	Asian	non-Hispanic	neg	Pos	neg	neg
P15	nd	nd	White	non-Hispanic	neg	neg	Pos	Pos
P16	F	35	Asian	non-Hispanic	Pos	Pos	neg	neg
P17	nd	nd	White	non-Hispanic	elevated	Pos	neg	neg
P18	M	67	White	non-Hispanic	neg	Pos	neg	neg
P19	F	53	White	non-Hispanic	neg	Pos	neg	neg
P20	nd	nd	White	non-Hispanic	neg	neg	Pos	Pos
P21	F	85	White	non-Hispanic	neg	elevated	neg	neg
P22	F	36	White	non-Hispanic	neg	Pos	neg	neg
P23	M	57	White	non-Hispanic	neg	Pos	neg	neg
P24	M	23	White	non-Hispanic	neg	neg	neg	neg
P25	F	52	White	non-Hispanic	neg	neg	neg	neg
P26	F	39	White	non-Hispanic	neg	neg	neg	neg
P27	M	32	White	non-Hispanic	neg	elevated	neg	neg
P28	M	79	Asian	non-Hispanic	neg	Pos	neg	neg
P29	M	47	Asian	non-Hispanic	neg	Pos	neg	neg
P30	M	65	White	non-Hispanic	neg	Pos	neg	neg
P31	F	45	White	non-Hispanic	neg	neg	neg	neg
P32	F	76	White	non-Hispanic	neg	elevated	neg	neg
P33	F	34	White	non-Hispanic	neg	Pos	neg	neg
P34	F	21	White	non-Hispanic	neg	Pos	neg	neg

Table S7. Odense University Hospital SLE cohort: clinical data of individual subjects showing positivity to antigens 9,11

Patient nr	Diagnosis	Manifestations	Anti-phospholipid analysis	Serology	Treatment
P3	Venous thropmboembolism, spinal stenosis, hyperlipidemia	Lumbal pain, pulmonary embolism	IgG a-b2GPI positive	None	Xarelto, Atorvastatin, Gabapentin
P6	Hepatocellulary carcinoma	Stomach pain, lumbal pain	IgM a-cardiolipin positive	None	None
P8	Urticarial vasculitis	Skin itch, thrombocytopenia	IgM a-b2GPI/a-cardiolipin positive	p-ANCA weak pos., ANA grade 5	Dapson
P12	None	Sclerotic aortic valve, TCI symptoms	IgG a-b2GPI positive	None	Lercanidipin, Atorvastatin, Clopidogrel, Losartan
P15	None	None	IgM a-cardiolipin pos.	None	None
P20	SLE	Arthritis, nephritis, cerebral apoplexia	IgG a-b2GPI positive	ANA-pos., DNA-Ab pos.	Marevan, Plendil, Simvastatin
