# Supplementary material for: Novel Phospholipid-Protein Conjugates Allow Improved Detection of Antibodies in Patients with Autoimmune Diseases
Source: PLoS One. 2016 Jun 3;11(6):e0156125. doi: 10.1371/journal.pone.0156125 (PMC4892602; doi:10.1371/journal.pone.0156125)
Supplement: S4 Appendix — (PDF) [file pone.0156125.s004.pdf]

S4 Appendix

Dose-response assay (Figure 2)

U/mL	A450 - Conjugate 9/ Patient nr. (SLE Stanford University Hospital cohort)							
	P2	P5	P7	P11	P13	P15	P18	P21
0,04	0,11	0,1	0,12	0,13	0,14	0,13	0,09	0,11
0,07	0,12	0,11	0,13	0,13	0,2	0,14	0,13	0,11
0,11	0,14	0,22	0,24	0,21	0,24	0,22	0,21	0,2
1,5	0,28	0,32	0,35	0,33	0,38	0,48	0,49	0,44
2,9	0,56	0,77	0,78	0,67	0,73	0,65	0,7	0,69
4,5	0,76	0,8	0,84	0,77	0,8	0,72	0,81	0,77
6,7	0,78	0,81	0,85	0,81	0,84	0,79	0,83	0,8

U/mL	A450 - Conjugate 11/ Patient nr. (SLE Stanford University Hospital cohort)							
	P2	P5	P7	P11	P13	P15	P18	P21
0,04	0,09	0,11	0,1	0,07	0,13	0,11	0,17	0,11
0,07	0,1	0,11	0,09	0,09	0,13	0,14	0,17	0,18
0,11	0,13	0,19	0,21	0,22	0,24	0,21	0,33	0,32
1,5	0,45	0,4	0,38	0,55	0,6	0,67	0,48	0,7
2,9	0,77	0,89	0,66	0,7	0,78	0,73	1,12	0,89
4,5	0,83	1,1	0,8	0,92	0,87	0,83	1,2	1,07
6,7	0,98	1,23	0,96	1,31	1,2	1,05	1,1	1,34

U/mL	A450 - β2GPI/ Patient nr. (SLE Stanford University Hospital cohort)							
	P2	P5	P7	P11	P13	P15	P18	P21
0,04	0,05	0,07	0,09	0,05	0,08	0,09	0,05	0,07
0,07	0,05	0,1	0,08	0,09	0,07	0,11	0,05	0,08
0,11	0,28	0,39	0,32	0,37	0,3	0,29	0,34	0,4
1,5	0,34	0,42	0,35	0,45	0,33	0,32	0,34	0,45
2,9	0,36	0,45	0,38	0,45	0,42	0,34	0,36	0,46
4,5	0,43	0,51	0,45	0,56	0,48	0,43	0,44	0,51
6,7	0,45	0,54	0,49	0,58	0,51	0,5	0,47	0,53

U/mL	A450 - β2GPI + CL/ Patient nr. (SLE Stanford University Hospital cohort)							
	P2	P5	P7	P11	P13	P15	P18	P21
0,04	0,44	0,36	0,4	0,52	0,41	0,48	0,54	0,38
0,07	0,51	0,49	0,56	0,66	0,55	0,59	0,61	0,56
0,11	0,67	0,68	0,8	0,72	0,63	0,6	0,72	0,68
1,5	0,77	0,72	0,85	0,78	0,83	0,74	0,8	0,87
2,9	0,84	0,78	0,89	0,78	0,94	0,9	1,1	0,94
4,5	0,91	0,89	0,93	0,99	1,11	1,04	1,18	1,14
6,7	0,89	0,92	0,94	0,89	1,17	1,1	1,11	1,17

Data Table - Figure 3A

Antigen/A450

b2GPI	PT	CL	PE	b2GPI+CL	PT+PE	8	9	10	11
0.15	0.11	0.55	0.11	0.08	0.57	0.14	0.14	0.11	0.06
0.16	0.17	0.55	0.12	0.08	**0.58**	0.15	0.14	0.16	0.07
0.18	0.20	0.55	0.14	0.09	0.58	0.15	0.15	0.20	0.07
0.20	0.23	0.56	0.14	0.10	0.60	0.19	0.18	0.20	0.10
0.20	0.25	0.57	0.16	0.12	0.61	0.22	0.21	0.23	0.12
0.20	0.30	0.57	0.18	0.14	0.62	0.26	0.22	0.23	0.12
0.20	0.32	0.57	0.19	0.15	0.62	0.26	0.24	0.24	0.12
0.21	0.33	0.57	0.20	0.17	0.62	0.28	0.24	0.28	0.12
0.22	0.35	0.58	0.21	0.18	0.62	0.33	0.26	0.41	0.14
0.23	0.36	0.59	0.26	0.18	0.63	0.33	0.26	0.50	0.16
0.24	0.36	0.60	0.26	0.18	0.63	0.34	0.27	0.51	0.17
0.24	0.39	0.60	0.32	0.19	0.63	0.35	0.28	0.52	0.18
0.25	0.56	0.61	0.39	0.19	0.64	0.39	0.28	0.56	0.18
0.27	0.56	0.61	0.57	0.20	0.67	0.39	0.29	0.57	0.20
0.41	0.57	0.66	0.57	0.23	0.70	0.41	0.29	0.57	0.20
0.66	0.75	0.69	0.60	0.28	0.71	0.45	0.30	0.59	0.24

Data Table - Figure 3B

Antigen/A450

b2GPI	***b2GPI+CL***	***9***	***11***
0.11	0.37	0.19	0.15
0.11	0.39	0.09	0.25
0.11	0.51	0.11	0.26
0.11	0.51	0.15	0.28
0.11	0.52	0.15	0.33
0.11	0.52	0.16	0.34
0.12	0.54	0.18	0.34
0.12	0.55	0.19	0.36
0.14	0.55	0.19	0.36
0.21	0.56	0.20	0.36
0.23	0.57	0.20	0.39
0.24	0.58	0.20	0.41
0.24	0.60	0.21	0.41
0.25	0.62	0.22	0.44
0.27	0.64	0.22	0.48
0.28	0.64	0.23	0.50
0.30	0.67	0.27	0.54
0.31	0.67	0.28	0.61
0.32	0.67	0.37	0.61
0.32	0.69	0.39	0.66
0.34	0.69	0.39	0.67
0.37	0.71	0.45	0.73
0.40	0.73	0.99	1.00
0.40	0.79	1.02	1.00
0.43	0.79	1.03	1.01
0.44	0.81	1.04	1.03
0.44	0.86	1.06	1.05
0.48	1.05	1.12	1.12

b2GPI	***b2GPI+CL***	***9***	***11***
0.17	0.29	0.15	0.21
0.21	0.21	0.22	0.28
0.23	0.21	0.23	0.28
0.25	0.22	0.23	0.29
0.56	0.66	0.66	0.30
0.26	0.22	0.26	0.32
0.26	0.22	0.26	0.33
0.28	0.23	0.26	0.34
0.28	0.25	0.27	0.35
0.28	0.27	1.28	0.35
0.29	0.33	0.32	0.36
0.30	0.35	0.33	0.36
0.33	0.51	0.38	0.38
0.34	0.55	0.38	0.39
0.34	0.55	0.41	0.40
0.57	0.61	0.44	0.40
0.35	0.55	0.42	0.42
0.37	0.57	0.55	0.44
0.92	0.69	0.87	0.45
0.63	0.66	0.66	0.45
0.76	0.66	1.16	0.45
0.38	0.57	0.59	0.46
0.39	0.57	0.60	0.48
0.41	0.57	0.60	0.49
0.41	0.57	0.60	0.49
0.42	0.57	0.64	0.50
0.42	0.57	0.64	0.50
0.44	0.58	0.71	0.50
0.52	0.59	0.83	0.54
0.52	0.59	0.83	0.54
0.57	0.61	0.50	0.60
0.53	0.60	0.56	0.69
0.53	0.60	0.87	0.69
0.58	0.61	0.50	0.70
0.55	0.60	0.30	0.72
0.55	0.60	0.76	0.72
0.40	0.69	0.80	0.76
0.26	0.22	0.25	1.10
0.44	0.58	0.71	0.50
0.40	0.69	1.29	0.76
0.52	0.59	0.83	0.54

Data Table - Figure 4

Smith pos/neg	A450
0	1.05
1	0.92
1	0.91
0	0.87
1	0.99
1	1.01
0	0.68
0	0.35
0	0.22
0	0.18
1	0.55
0	0.44
0	0.26
1	0.62
1	0.77
0	0.44
1	0.72
0	0.48
1	0.74
0	0.34
0	0.36
1	0.39
0	0.62
0	0.22
